# Male Senescence Phenotypes Include Hair Loss and Hair Gain

**DOI:** 10.7759/cureus.115552

**Published:** 2026-08-31

**Authors:** Colleen Reisz, An-Lin Cheng

**Affiliations:** 1 Internal Medicine, University of Missouri-Kansas City School of Medicine, Kansas City, USA; 2 Biomedical and Health Informatics, University of Missouri-Kansas City School of Medicine, Kansas City, USA

**Keywords:** auditory science, conchal hair growth rating scale, developmental origins of adult disease theory, geroscience, male pattern baldness, maternal-fetal interface

## Abstract

Background: Male pattern baldness is a prominent component of senescence phenotypes. Hair loss on the scalp is frequently accompanied by hair gain in the concha. The change in hair follicle fate near sensory apertures, such as the ear, nose, and eye, may be more than a cosmetic concern and represent an age-related adaptation in neurosensory communication. Epidemiologists have proposed that the propensity for age-related diseases is programmed during gestation.

Objectives: This study aimed to investigate variables related to the maternal-fetal interface to hair loss and hair gain measurements in men, as well as to investigate the relationship between age-related hair loss on the scalp and concurrent hair gain in the concha in men.

Methods: This is a cross-sectional observational study conducted in a general dermatology clinic. Because accurate birth data are not available in this study population, three proxy variables (maternal age, birth order, and intersibling gap measured in months) were used to approximate the intrauterine environment on follicle fate. Hamilton Norwood ratings were collected by a dermatologist. Historical recall on height and weight at age 18 was compared with actual height and weight collected at the time of visit.

Results: Significant negative correlations were observed between maternal age and intersibling gap, and hair loss scores. Higher hair loss scores on the Hamilton-Norwood scale correlated directly with hair gain in the conchal bowl.

Conclusions: The gain of whisker-type hair in the conchal bowl, a key sensory aperture, may have clinical applications outside cosmetic concerns. The conchal hair growth scale may have greater inter-rater reliability across disciplines that study aging.

## Introduction

Rather than treating disease, scientists in the field of aging aim to minimize cellular damage. Geroscience, the multidisciplinary field of science that studies aging, emphasizes health span, the amount of time that people stay healthy [1,2]. Dermatologists participate in anti-aging research, diagnosing and treating one of the earliest clinical features of aging, male pattern baldness (MPB). MPB has associations with other age-related diseases, such as diabetes and cardiovascular diseases [3,4]. The connections between follicle health and chronic disease have led to a greater understanding of signaling pathways that start during embryogenesis and continue through life [5]. Signals for hair growth and regeneration utilize crosstalk between Wnt/b catenin, sonic hedgehog, bone morphogenetic proteins, and Notch, all of which are present during embryogenesis [6]. Age, insulin, nutrient excess, and androgens alter the perifollicular dermal fat and surrounding mesenchyme that support hair growth and regeneration [7]. Recent studies on MPB have revealed complex genetics and an association with the X chromosome [8,9]. In this study, hair follicle populations were quantified in two regions, the scalp and the concha.

In addition to the field of geroscience, epidemiologists have identified the maternal-fetal interface as a key programming influence for age-related diseases [10]. Early maternal age has been associated with prematurity and intrauterine growth retardation [11]. The Developmental Origins of Adult Disease (DOHaD) theory continues to drive improvements in obstetric care, as children with adverse gestational environments are known to have higher risks of metabolic diseases [12]. Low-birth-weight babies develop a "thrifty phenotype," which expresses itself as insulin resistance. For this study, accurate birth data were not available for the study cohort, who were born prior to the introduction of electronic medical records. To address the lack of accurate birth data, surrogate markers were used to study the relationship between the maternal-fetal interface and male pattern baldness. Maternal age, birth order, and intersibling gap in months were collected from the male participants in this study.

Hair loss was graded using the Hamilton-Norwood scale, introduced in the 1950s by Dr. Hamilton and refined by Dr. Norwood in the 1970s [13]. This validated scoring system does not address hair gain at the ear, so a scoring system was designed for this study, the Conchal Hair Growth Rating Scale. The concha directs sound waves to the eardrum, allowing the conversion of vibration to neural signals that reach the cochlea [14-16]. The mechanisms that connect hair movement and deflection with neural uptake have been well studied in rodents, prompting interest in the possibility of hair gain in the concha as an age-related adaptation.

## Materials and methods

The study involved a cross-sectional observation of 474 male participants from a general dermatology clinic in the Kansas City metropolitan area. Sensitivity analyses were performed to validate the significant associations identified through Pearson's correlation with regression modeling adjusted for age. Collected variables included age, race, and current height and weight. Patients were asked to recall their tallest height and corresponding weight, from which a historical body mass index (BMI) was calculated. The historical and current BMI calculations were compared, and a delta value was calculated. Maternal-fetal interface proxy measures included maternal age at birth, birth order, and interval in months to the next oldest sibling (for participants who were not firstborn). To assess and quantify scalp hair growth patterns, a single board-certified dermatologist used the Hamilton-Norwood scoring system, a validated hair loss scale specifically designed for men (Figure 1). In addition to the assignment of a male pattern baldness score, participants were examined for whisker-like hair growth in the concha. The Conchal Hair Growth Rating Scale ranked growth from 1 to 3, with a score of 1 assigned to those with no observable whisker hair growth (Figure 2), a score of 2 for those with 8 or fewer terminal hairs per ear (Figure 3), and a score of 3 for greater than 9 terminal hairs per ear (Figure 4).

**Figure 1:**
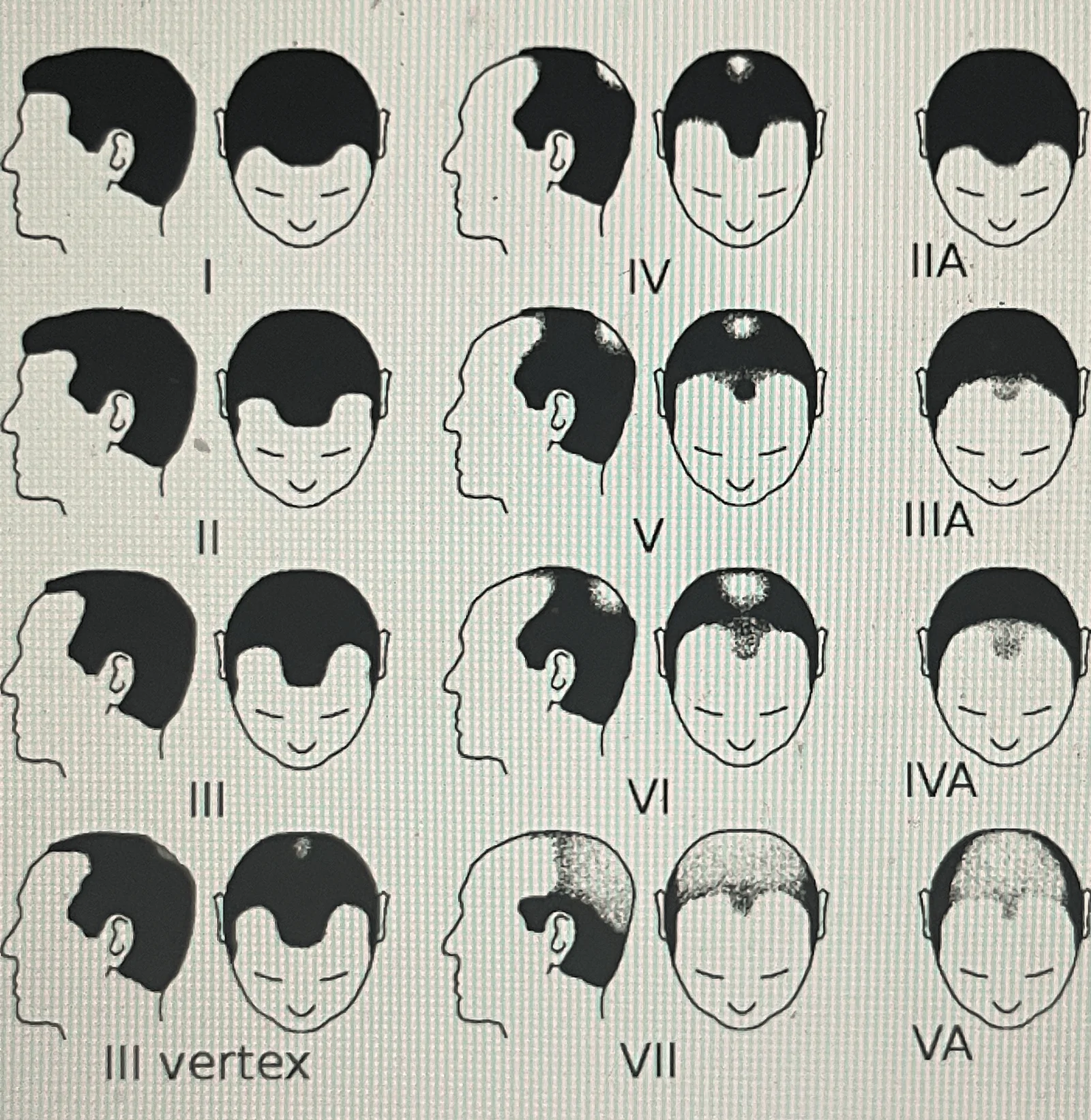
Hamilton-Norwood Scale Source: [13]

**Figure 2:**
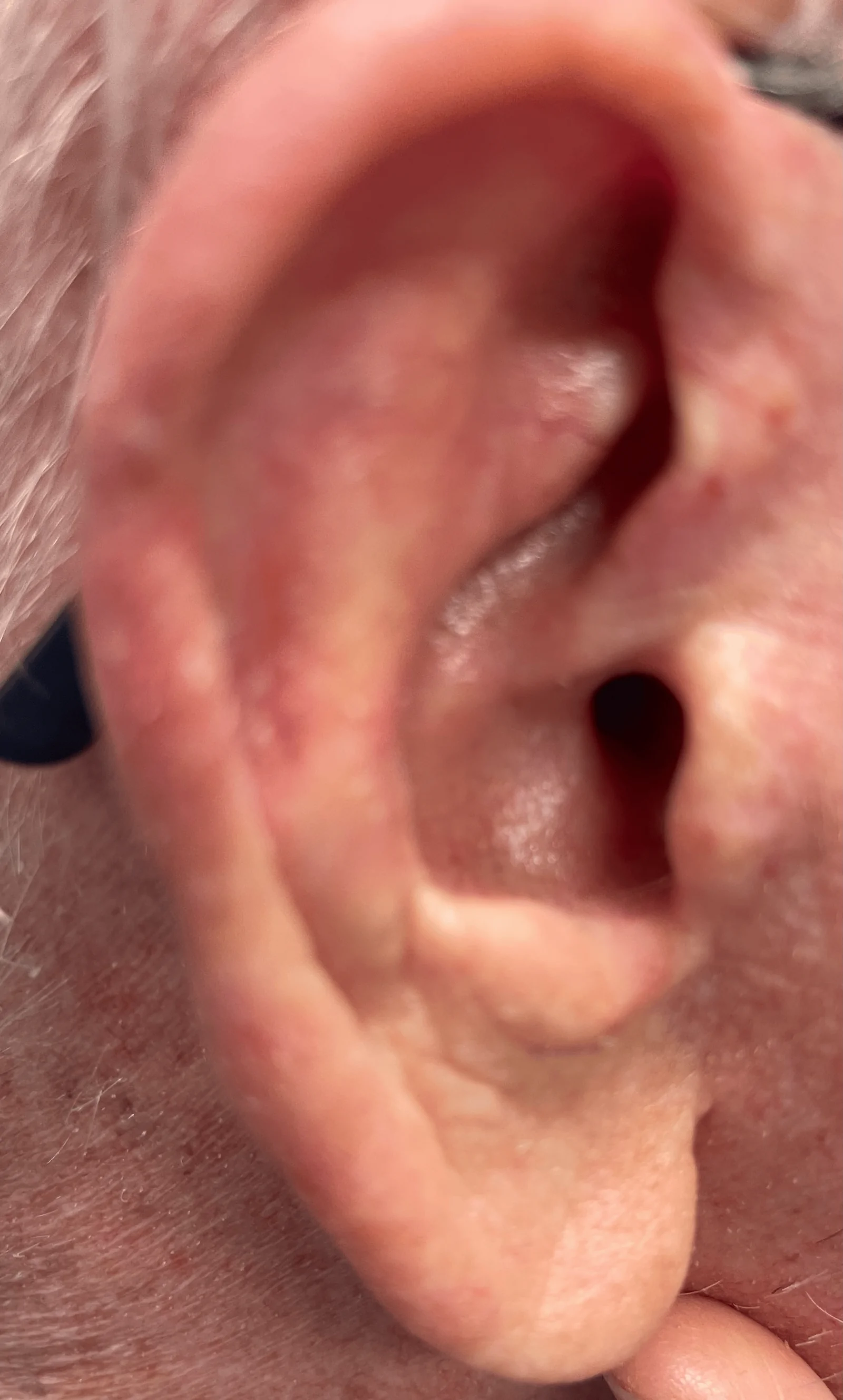
Conchal Hair Growth Scale 1 Right conchal bowl without terminal hair growth Permission received from the patient

**Figure 3:**
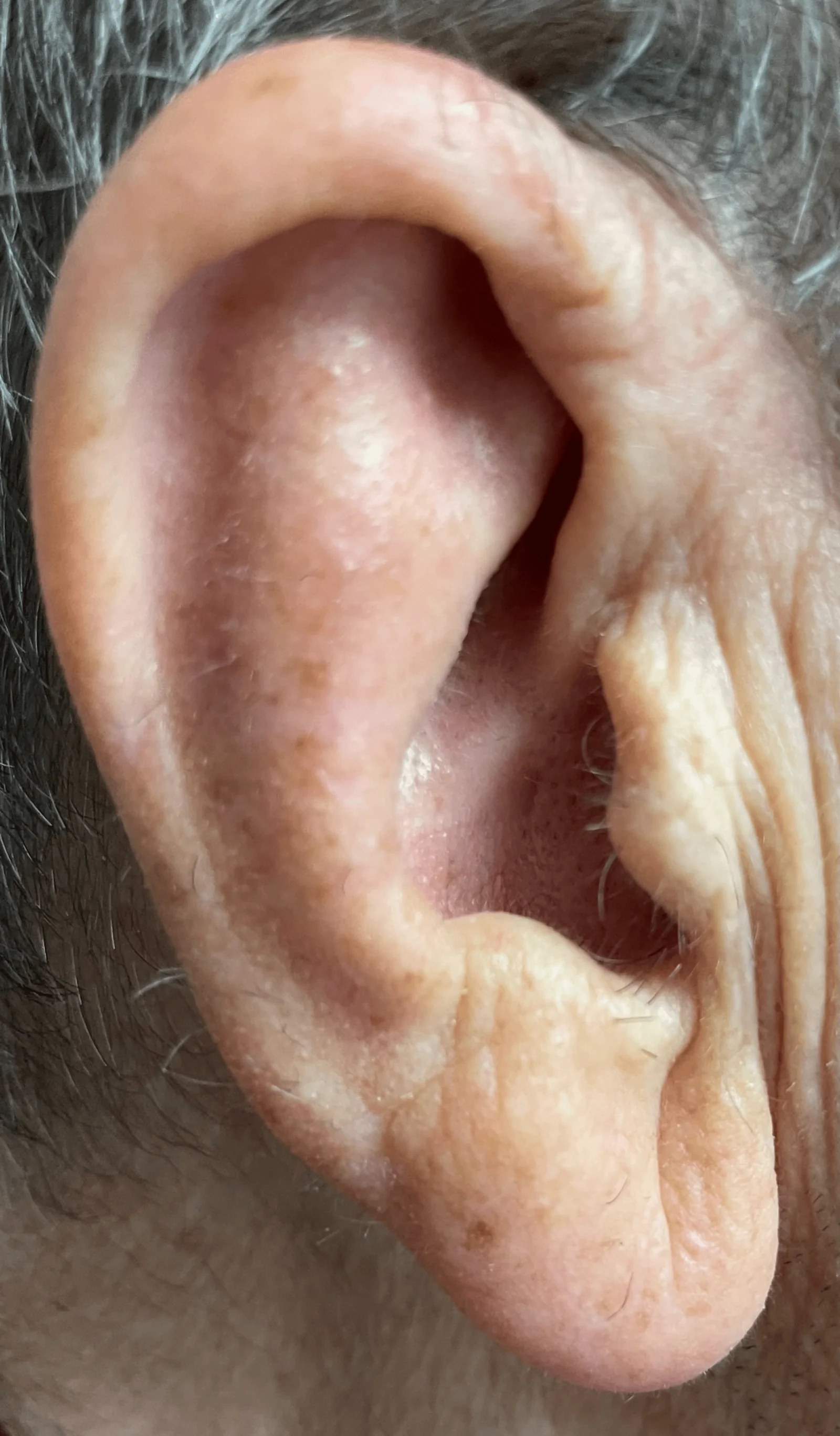
Conchal Hair Growth Scale 2 Right conchal bowl with level 2 terminal hair growth Permission received from the patient

**Figure 4:**
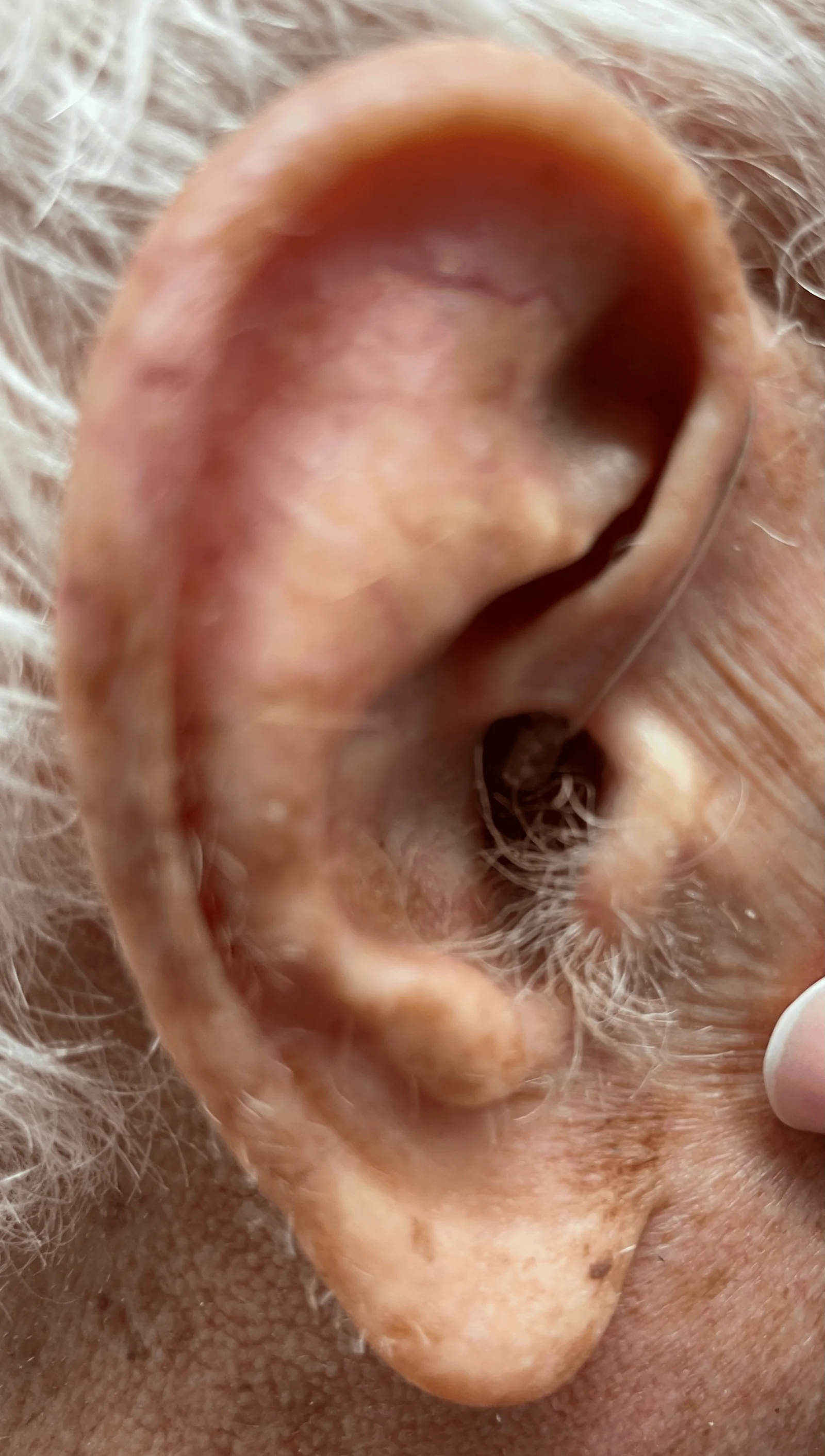
Conchal Hair Growth Scale 3 Right conchal bowl with level 3 of terminal hair growth Permission received from the patient

Figure 5 presents an illustration of the human ear anatomy showing the relationship between the external auditory canal and auditory pathways in the brain.

**Figure 5:**
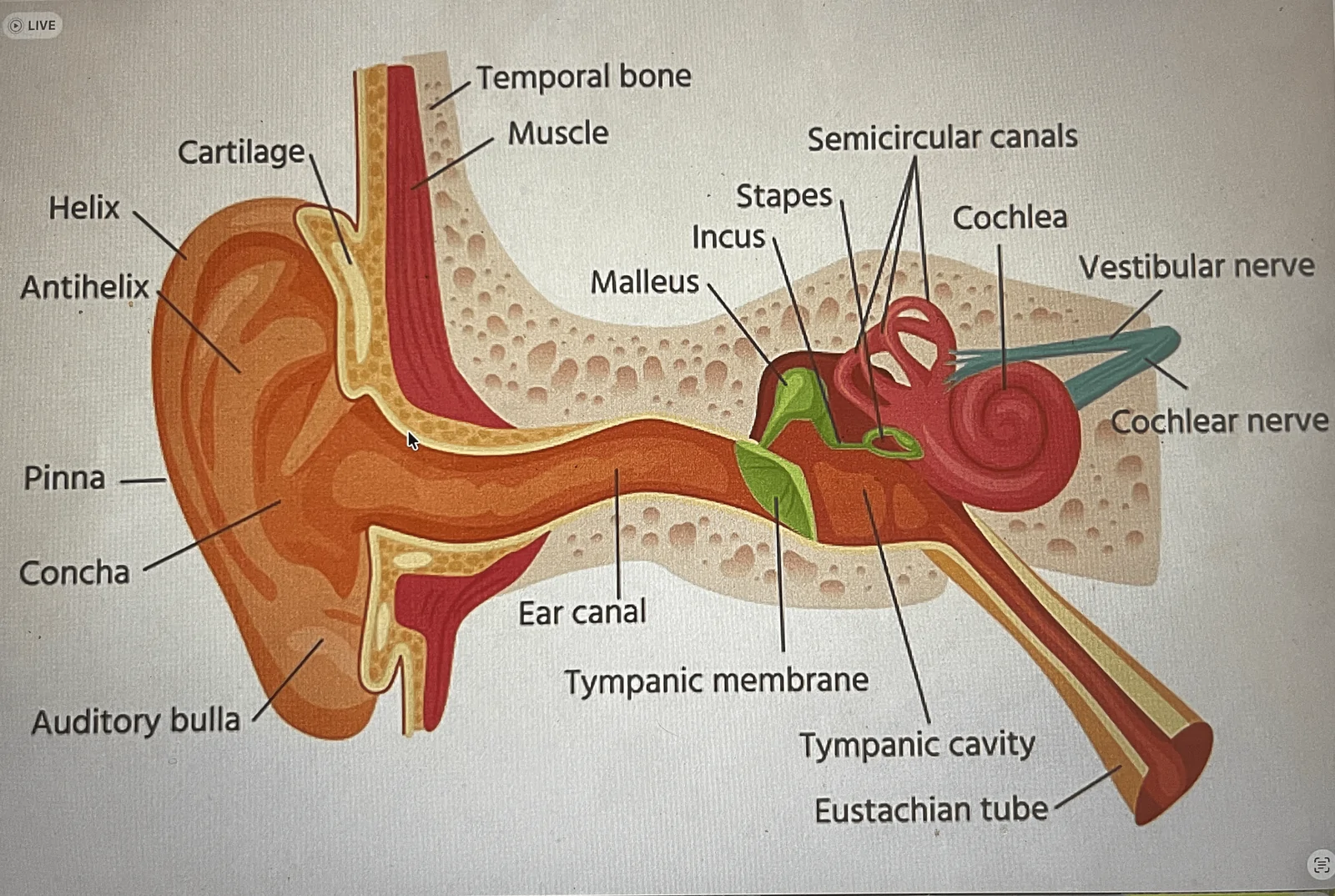
Human ear anatomy illustrating the relationship between the external auditory canal and auditory pathways in the brain The concha directs vibrations from sound waves toward the tympanic membrane. The hair cells in the inner ear convert the vibrations to electrical signals. The signals are received by the cochlea, which is the first central auditory nucleus. Source: iStock, Tetiana Lazunova

## Results

Data were collected from 474 male subjects, aged 45-94 years (mean: 69 years). The average maternal age at the time of birth was 27.4 years, and subjects had an average of 3.8 siblings. For non-first-born participants, the mean interval between their birth and that of their next oldest sibling was 25 months.

Pearson's correlation coefficient was used with two regression models run to examine the association between maternal age and intersibling gap while accounting for age. The beta coefficients were as follows: Hamilton-Norwood stage of hair loss and maternal age (β = -0.0363, p = 0.035) (Figure 6), Hamilton-Norwood stage and intersibling gap (β = -0.010, p = 0.0055) (Figure 7), and Hamilton-Norwood stage and conchal hair growth (β = 0.2243, p = 0.0059).

**Figure 6:**
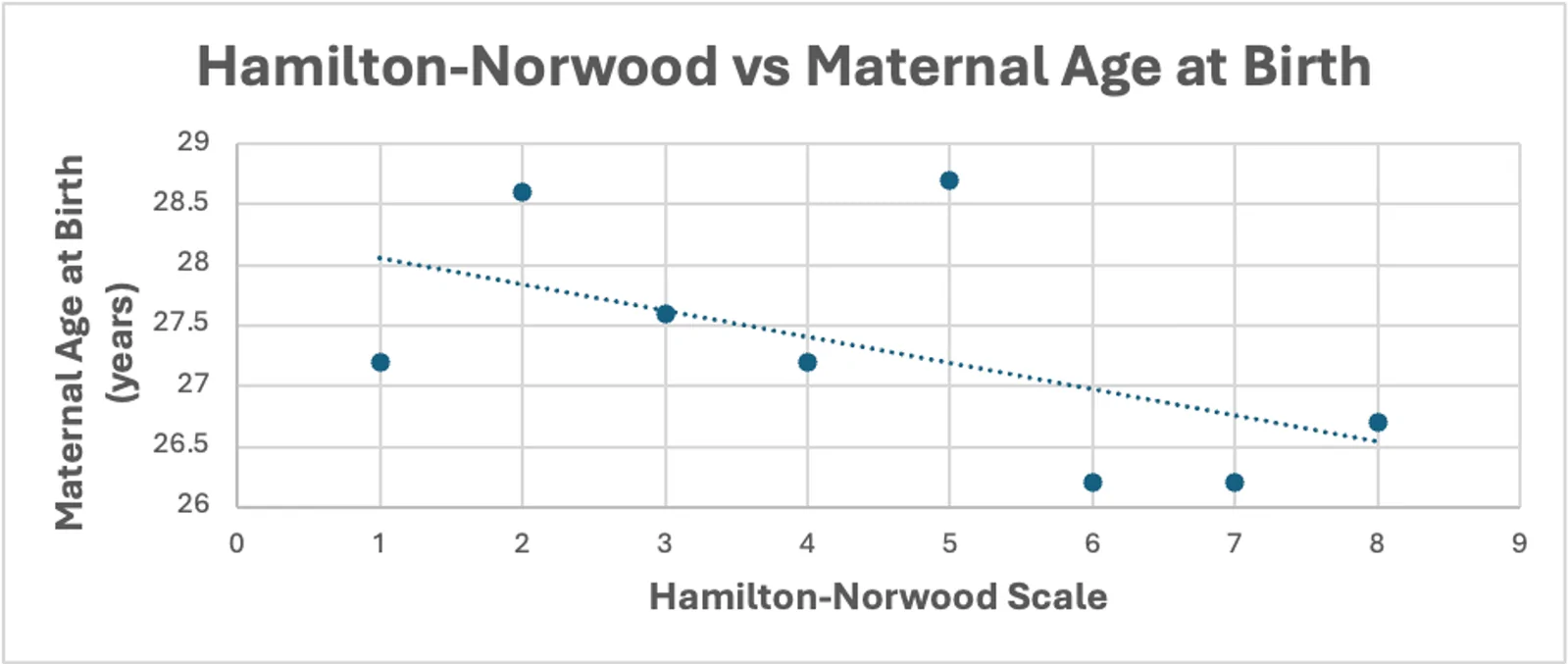
Hamilton-Norwood Scale Versus Average Maternal Age A negative correlation was seen between maternal age and hair loss scores in adulthood (β = -0.0363, p = 0.035).

**Figure 7:**
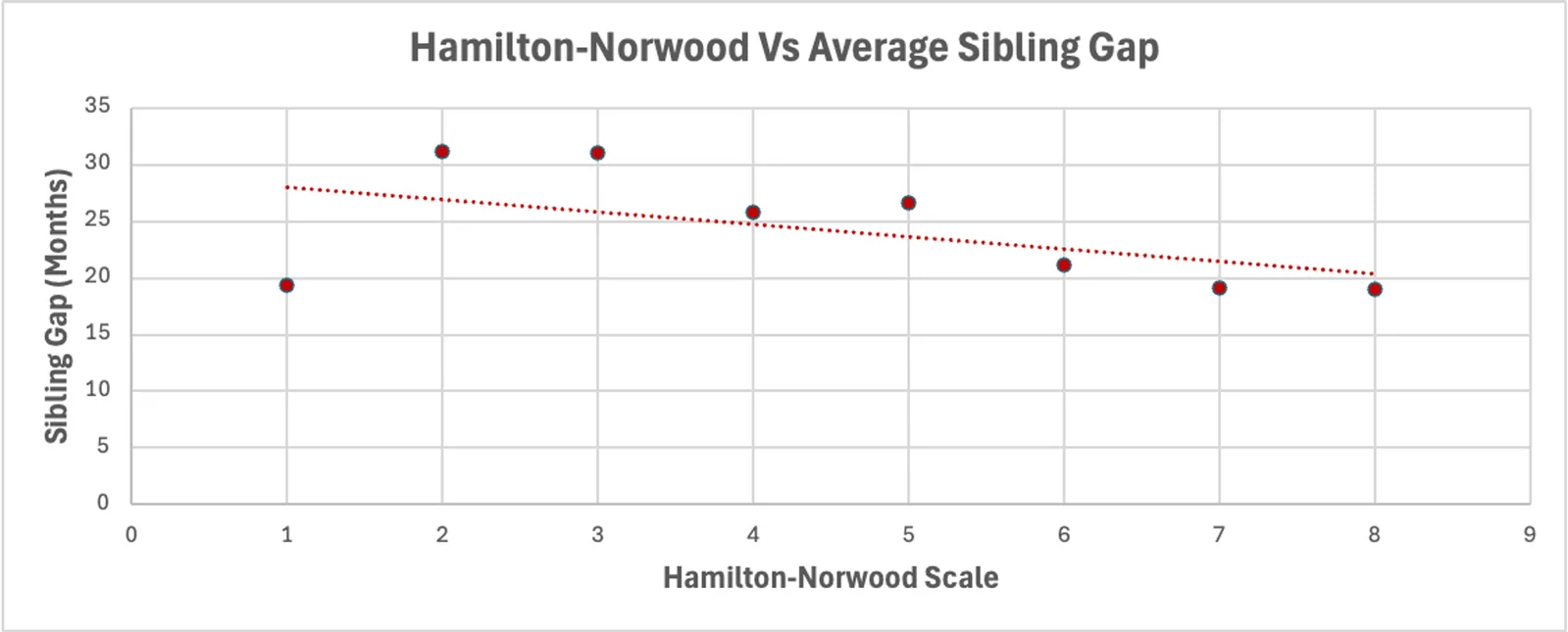
Hamilton-Norwood Scale Versus Average Sibling Gap Excluding the 167 (35%) participants who were firstborns, participants with smaller intersibling gaps exhibited more severe hair loss compared to those with larger intersibling gaps (β = -0.010, p = 0.0055).

The patient's recall of tallest height with corresponding weight was compared to actual measurements of their height and weight at the time of data collection. Among the 42 (9%) participants noted to have a reduction in BMI, height loss was found in 23/42 (55%). The average age of those who lost height with loss of BMI was 77, with two outliers, age 56 and 57. Both outliers had older mothers (29 and 34), were the youngest in sibling order (3/3 and 5/5), and had Hamilton-Norwood/Conchal Hair Growth scores of 2 and 3, and 2 and 2, respectively. Height loss was also noted in 45% of those whose BMI increased, so the weight component of the BMI calculation obscured the loss in height. As the BMI calculation is still commonly used, a reduction in BMI may be the broader safety signal for height loss (55%/45%).

## Discussion

Hair follicles are constantly changing through life. The lanugo hair of infancy gives way to terminal hair growth on the scalp. Follicles in the axilla, beard, and pubic area undergo dramatic changes in texture and density at puberty. Hormones continue to influence hair growth through adolescence and early adulthood, the effects of which start to wither in the late 20s and 30s. Hair follicles function as a conduit for oil and sweat, providing moisture, reducing friction, and serving a role in reproduction through pheromones [17,18].

Although the follicles on the scalp and the concha have been present since birth, the growth potential changes with age. Fatty liver realigns embryologic patterning genes, altering the placement, growth, and regression cycles of the hair follicle [7,19]. Hair on the scalp miniaturizes and becomes vellus while follicles in the concha go in the opposite direction, becoming coarse and curly, with comedones and visible oil production. Hair follicles have mechanical properties and respond to deflection and air movement [16,20]. Hair in the concha, one considered a cosmetic concern, may be an adaptation to age-related hearing loss or vestibular function.

The significant findings in this study, that higher hair loss scores are seen in men born to younger mothers and those with shorter intersibling gaps, support the hypothesis that the gestational environment programs the propensity for adult diseases, such as MPB. Maternal age has been associated with different pregnancy outcomes. Children born to young mothers have a greater risk of low birth weight and tend toward a thrifty phenotype and insulin resistance [17].

Lastly, hair loss scores in this cohort were directly correlated with hair growth scores in the concha, suggesting similar biologic mechanisms. The mesenchymal signal mix that underlies hair loss on the crown of the scalp may have a different effect on hair growth in the concha. The Hamilton-Norwood scale is a validated hair loss scoring system well known in dermatology and cosmetic hair restoration fields. This scale does not address hair growth at the concha, which may be a simpler measure of age-related changes to the hair follicle that could be adapted across all disciplines that study aging [21,22].

The secondary outcome measures, including changes in BMI and height, illustrate the limitations of BMI as a sole indicator of body composition changes, particularly in older individuals. A significant number of participants experienced height loss despite an increase or no change in BMI.

The cross-sectional design of the study and the sample from a multi-site practice across a large metropolitan area in the Midwest may limit the generalizability of our findings. Specifically, less than 2% of the participants identified as non-white, limiting the application of our results across diverse racial and ethnic groups. Reliance on self-reported data introduces the possibility of recall and desirability bias. The use of BMI as a measure does not differentiate between relative losses or gains in fat, bone, and muscle, leading to inaccuracies in assessing body composition changes. Moreover, our study did not control for confounding variables such as concurrent medication use, which could influence MPB onset and progression. Lastly, the dermatologist collecting data was not blinded to the study's objectives, introducing possible bias in the results interpretation.

## Conclusions

Male pattern baldness (MPB) has known associations with metabolic diseases. As proposed by the Developmental Origins of Adult Disease (DOHaD), the propensity for metabolic diseases in adulthood is programmed during gestation. Hair follicle growth cycles are governed by programming genes present during gestation. Hair loss and hair gain profiles in older men may be clinical expressions of embryologic patterning genes that have been altered by age and fatty liver.
